# Macrophage depletion in stellate ganglia alleviates cardiac sympathetic overactivation and ventricular arrhythmogenesis by attenuating neuroinflammation in heart failure

**DOI:** 10.1007/s00395-021-00871-x

**Published:** 2021-04-21

**Authors:** Dongze Zhang, Wenfeng Hu, Huiyin Tu, Bryan T. Hackfort, Bin Duan, Wanfen Xiong, Michael C. Wadman, Yu-Long Li

**Affiliations:** 1 Department of Emergency Medicine, University of Nebraska Medical Center, Omaha, NE 68198 USA; 2 Department of Cellular and Integrative Physiology, University of Nebraska Medical Center, Omaha, NE 68198 USA; 3 Mary & Dick Holland Regenerative Medicine Program, Division of Cardiology, Department of Internal Medicine, University of Nebraska Medical Center, Omaha, NE 68198 USA; 4 Department of Surgery, University of Nebraska Medical Center, Omaha, NE 68198 USA

**Keywords:** Macrophage depletion, Clodronate liposomes, Calcium channel, Cardiac sympathetic neuron, Ventricular arrhythmia, Chronic heart failure

## Abstract

Cardiac sympathetic overactivation is involved in arrhythmogenesis in patients with chronic heart failure (CHF). Inflammatory infiltration in the stellate ganglion (SG) is a critical factor for cardiac sympathoexcitation in patients with ventricular arrhythmias. This study aims to investigate if macrophage depletion in SGs decreases cardiac sympathetic overactivation and ventricular arrhythmogenesis in CHF. Surgical ligation of the coronary artery was used for induction of CHF. Clodronate liposomes were microinjected into bilateral SGs of CHF rats for macrophage depletion. Using cytokine array, immunofluorescence staining, and Western blot analysis, we found that macrophage expansion and expression of TNFα and IL-1β in SGs were markedly increased in CHF rats. Flow cytometry data confirmed that the percentage of macrophages in SGs was higher in CHF rats than that in sham rats. Clodronate liposomes significantly reduced CHF-elevated proinflammatory cytokine levels and macrophage expansion in SGs. Clodronate liposomes also reduced CHF-increased N-type Ca^2+^ currents and excitability of cardiac sympathetic postganglionic neurons and inhibited CHF-enhanced cardiac sympathetic nerve activity. ECG data from 24-h, continuous telemetry recording in conscious rats demonstrated that clodronate liposomes not only restored CHF-induced heterogeneity of ventricular electrical activities, but also decreased the incidence and duration of ventricular tachycardia/fibrillation in CHF. Macrophage depletion with clodronate liposomes attenuated CHF-induced cardiac sympathetic overactivation and ventricular arrhythmias through reduction of macrophage expansion and neuroinflammation in SGs.

**Supplementary Information:**

The online version contains supplementary material available at 10.1007/s00395-021-00871-x.

## Introduction

As a common complication in chronic heart failure (CHF), ventricular arrhythmia accounts for nearly 50–60% of mortality in patients with CHF [9]. Besides structural and electrophysiological remodeling in the ventricle [49], neuronal remodeling in the autonomic nervous system plays an important role in the development and maintenance of ventricular arrhythmias in CHF [36]. It has been demonstrated that excess cardiac sympathoexciation with the subsequent release of neurotransmitters from nerve terminals is a substrate to evoke ventricular arrhythmias in CHF [9, 25]. However, the management of sympathetic overactivation remains challenging in the clinic. This study attempted to explore a novel therapeutic strategy to inhibit cardiac sympathetic overactivation and ventricular arrhythmias.

Recent studies have revealed an elevation of inflammatory infiltration and macrophage hyperactivation in stellate ganglion (SG) from patients with cardiomyopathy and arrhythmias [1, 40]. It remains unclear if and how neuroinflammation in the SG contributes to ventricular arrhythmias. Voltage-dependent calcium channels (VDCCs) play an important role in a wide variety of cell functions through mediating calcium influx [20]. Among various types of VDCCs, N-type calcium (Cav2.2) channels, predominantly expressed in the nervous system, are considered to be essential for modulating neurotransmitter release at sympathetic nerve terminals [20, 32]. Our recent study has reported that N-type Ca^2+^ currents and excitability of cardiac sympathetic postganglionic (CSP) neurons located in the SGs are enhanced in CHF rats [58]. In the meantime, cardiac sympathetic overactivation was accompanied with lethal ventricular arrhythmias in the same CHF model [58]. However, the mechanisms underlying CHF-increased N-type Ca^2+^ currents and excitability of CSP neurons in the SGs are still unclear. Considering that voltage-gated Ca^2+^ channels could be modulated by proinflammatory cytokines in various tissues and cells [43, 53], it is possible that CHF-enhanced N-type Ca^2+^ currents in CSP neurons and cardiac sympathoexcitation are attributed to neuroinflammation in the SGs.

Proinflammatory cytokines are produced predominantly by activated macrophages [14]. Depleting macrophages allows researchers to investigate their functions by observing the consequences of cell absence [19]. Clodronate liposomes, phagocytized by macrophages to induce macrophage apoptosis and depletion [42], have been chosen to eliminate macrophages in many studies [21, 52, 55]. By employing clodronate liposomes, some studies emphasized the critical role of brain macrophages in systemic inflammation-triggered central sympathetic excitation in rats with acute myocardial infarction (MI) [55] and hypertension [21]. Thus far, there is no available information about the contribution of neuroinflammation in SGs to cardiac sympathetic overactivation and ventricular arrhythmias in CHF. In the present study, therefore, we microinjected clodronate liposomes into the SGs of CHF rats to test whether local depletion of macrophages in SGs reduces N-type Ca^2+^ currents and excitability of CSP neurons and subsequently decreases cardiac sympathetic overactivation and ventricular arrhythmogenesis in CHF.

## Methods

The study conformed to guidelines for the Care and Use of Laboratory Animals and was approved by the Institutional Animal Care and Use Committee (IACUC, NO.18-070-06-FC) at the University of Nebraska Medical Center. See supplemental materials for detailed description in methods.

### Experimental design

In the present study, a total of 152 male Sprague–Dawley rats (6–7 weeks of age, 180–200 g) were used and they were randomly assigned to sham or CHF group. CHF rats underwent surgical left anterior descending coronary artery (LAD) ligation for induction of MI-related CHF, and sham rats underwent the same surgery without LAD ligation. Implantation of radiotelemetry and labeling of CSP neurons were performed at 11 weeks post-MI. Then, CHF rats were divided into three subgroups for different treatments, including CHF, CHF treated with PBS liposomes, and CHF treated with clodronate liposomes at 12 weeks post-MI. PBS liposomes or clodronate liposomes were microinjected into bilateral SGs. Heart rate variability (HRV), ventricular arrhythmogenesis-related ECG makers, and spontaneous ventricular arrhythmias from 24-h radiotelemetry ECG recording in conscious rats were evaluated in all groups. Terminal experiments including measurement for inducibility of ventricular arrhythmia and cardiac sympathetic nerve activity (CSNA) recording in anesthetized rats, hemodynamic and morphological measurements, echocardiography, Western blot, immunofluorescence staining, cytokine array, flow cytometry, and whole-cell patch-clamp recording in CSP neurons were performed at 13–14 weeks post-MI (Supplemental Fig. 1).

### Animal model

CHF rats were anesthetized with 2% isoflurane for surgical ligation of the LAD, and sham rats underwent the same surgery without LAD ligation, as described previously [58, 59]. A Millar pressure transducer was used to determine left ventricle end-diastolic pressure (LVEDP) and systolic pressure (LVSP) in the terminal experiment. CHF was confirmed by multiple morphological and hemodynamic parameters (Table 1).

**Table 1:** Hemodynamic and morphological characteristics in all groups of rats

	Sham (*n* = 35)	CHF (*n* = 35)	CHF + PBS liposomes (*n* = 30)	CHF + Clodronate liposomes (*n* = 30)
Body weight (g)	411.83 ± 2.33	410.29 ± 2.73	409.09 ± 3.20	413.23 ± 3.16
MBP (mmHg)	101.94 ± 2.04	101.46 ± 2.15	102.13 ± 1.87	103.90 ± 2.25
HR (bpm)	354.46 ± 4.94	359.60 ± 5.08	358.73 ± 5.61	356.97 ± 6.12
Infarct size (% of LV)	0	45.34 ± 0.63*	45.47 ± 0.79*	45.33 ± 0.93*
LVSP (mmHg)	122.06 ± 1.56	98.77 ± 1.69*	96.30 ± 1.42*	96.27 ± 1.67*
LVEDP (mmHg)	1.71 ± 0.11	19.50 ± 0.47*	19.83 ± 0.48*	19.68 ± 0.50*

Data are means ± SE

*CHF* chronic heart failure, *MBP* mean blood pressure, *HR* heart rate, *LV* left ventricle, *LVSP* left ventricular end-systolic pressure, *LVEDP* left ventricular end-diastolic pressure

*p < 0.05 vs. Sham

### In vivo microinjection of clodronate liposomes into SGs

After SGs were identified, clodronate liposomes (Clophosome, 2 µl, 20 mg/ml, CAT: F70101C-AH, FormuMax Scientific Inc, Sunnyvale, CA, USA) or PBS liposomes (CAT: F70101-AH) were microinjected into the bilateral SGs by a glass micropipette. The terminal experiments were performed at 1 week after microinjection.

### Implantation of the ECG telemeter and ECG recording in conscious rats

Implantation of the ECG telemeter was performed as described previously [3, 38, 46]. Under the anesthetized condition, laparotomy was performed at the Linea Alba. The ECG transmitter was placed into the abdominal cavity and bipolar electrodes were tunneled subcutaneously. The negative and positive electrodes were secured in underlying tissue near the upper sternal midline and the left side of the xiphoid process, respectively. One week after ECG telemeter implantation, 24-h, continuous ECG recording was performed in conscious rats to quantify the ventricular arrhythmic events including premature ventricular contractions (PVCs) and the ventricular tachycardia/fibrillation (VT/VF). QT and corrected QT (QTc) intervals, as well as T-peak to T-end (Tpe) interval, were calculated from ECG segments in conscious rats.

### Measurement of the HRV in conscious rats

HRV measurement was used to compare sympathetic activation in conscious rats in the current study. For quantification of the HRV, 24-h, continuous ECG signals were acquired in conscious rats. The HRV was analyzed from ECG segments during the 24 h recording in conscious rats. HRV analysis including low frequency power (LF) from 0.2–0.75 Hz, high frequency power (HF) from 0.75–2.5 Hz, and LF/HF ratio was performed in current study [5, 34, 41].

### Labeling of SG neurons and whole cell patch-clamp recording for Ca^2+^ currents and action potentials (APs)

Considering that SG neurons project to the heart and other target organs, a transported fluorescent dye was used to retrogradely label SG neurons projecting to the myocardium, as described previously [39, 58]. The isolation of SG neurons for patch-clamp recording was performed at least 1 week after surgery to allow the dye to diffuse to SG neurons. SG neurons were isolated by a two-step enzymatic digestion protocol as described previously [50, 58]. Only DiI-labeled SG neurons (i.e. CSP neurons) were used to record voltage-gated Ca^2+^ currents and APs through the whole cell patch-clamp technique [58]. When the holding potential was − 80 mV, a 5-mV step increment between − 60 mV and 60 mV for 500 ms was used to elicit current–voltage (I−V) relationships. A saturating concentration of ω-conotoxin GVIA (1 μM) [23, 50, 58] was used to block N-type Ca^2+^ channels in the present study. N-type Ca^2+^ currents were calculated using an equation: N-type Ca^2+^ currents = total Ca^2+^ currents − Ca^2+^ currents under treatment of ω-conotoxin GVIA [50, 58]. In current-clamp experiments, a current injection of 100 pA was used to elicit APs, and frequency of APs was measured in a 1-s current clamp.

### Measurement of inducibility of ventricular tachyarrhythmia in anesthetized rats

Surface lead-II ECG was recorded through subcutaneous electrodes under the anesthetized condition. Programmed electrical stimulation (PES) was performed to determine the susceptibility to ventricular tachyarrhythmia in anesthetized rats. A programmed stimulation protocol combined by single (S2), double (S3), or triple extra-stimulus (S4) after a train of eight stimuli (8 × S1) was designed to induce ventricular tachyarrhythmia as described previously [18, 24, 46]. A quotient of ventricular arrhythmia score was used to quantify inducibility of ventricular tachyarrhythmia as described previously [24, 35].

### Recording of CSNA in anesthetized rats

CSNA was recorded in anesthetized rats as described previously [57, 58]. The left thoracotomy was performed in the second intercostal space and then the left cardiac sympathetic nerve was exposed and dissected distal to the left SG. The central cut end of the cardiac sympathetic nerve was placed on a bipolar platinum electrode for CSNA recording.

### Measurement of hemodynamic and morphological parameters

Under the anesthetized condition, the right femoral vein and left femoral artery were cannulated for the administration of drugs and monitoring blood pressure and heart rate, respectively. A Millar pressure transducer was inserted into the left ventricle for measurement of LVSP and LVEDP. After in vivo experiments were performed, the rat heart was isolated for measurement of infarct size through a colorimetric technique coupled to a computerized planimetric analysis.

### Immunofluorescence staining and Western blot analysis

For immunofluorescence staining, isolated SGs were fixed and cut into 10 μm-thick sections at -20 °C and then successively incubated with 10% donkey serum, primary antibodies, and appropriate secondary antibodies for observing cytokine imaging under a laser scanning confocal microscope. For Western blot analysis, protein level was measured using primary antibodies against a target or housekeeping protein and appropriate secondary antibodies. Target protein was normalized by housekeeping protein. Immunofluorescence staining and Western blot analysis were used to determine protein expression of ionized calcium-binding adaptor molecule 1 (Iba1), tumor necrosis factor alpha (TNFα), and interleukin-1 beta (IL-1β) in SGs.

### Cytokine protein array

A Rat Cytokine Antibody Array C1 kit was used to screen the levels of cytokine in SGs according to the User Manual. Briefly, the SG sample was added on an array membrane and successively incubated with biotin-conjugated antibody and infrared fluorescent dye-conjugated streptavidin. Finally, the washed membrane was scanned by the infrared image system.

### Flow cytometry analyses for macrophage infiltration

Flow cytometry is a widely used approach for measuring macrophage infiltration or expansion in many studies [28, 29, 47]. Isolated live cells from SGs obtained by enzymatical digestion were incubated with a mix of fluorochrome-conjugated antibodies against CD45 and CD11b and then analyzed by a LSRII cell analyzer. Macrophages were identified as CD45^+^/CD11b^+^ cells [6, 15, 45].

### Echocardiography

Echocardiography was performed to determine cardiac function [58]. Briefly, B-mode images were acquired in the parasternal long axis under light isoflurane anesthesia. M-mode images were acquired at the level of the left ventricular papillary muscles. Left ventricular end-diastolic diameter (LVDd) and left ventricular end-systolic diameter (LVDs) were measured. Then ejection fraction (EF), fractional shortening (FS), left ventricular end-diastolic volume (LVd Vol), and left ventricular end-systolic volume (LVs Vol) were calculated using standard formulas from the VisualSonics VevoLab software.

### Statistical analysis

All data are presented as means ± SEM. SigmaPlot 12 was used for data analysis. A student’s unpaired *t*-test was used to compare cytokine levels between two groups. A Chi-Square test was used to analyze incidence of ventricular arrhythmias. One-way ANOVA with post-hoc Bonferroni test was used to complete multi-group comparison for other measurements designed in this study. Normal distribution of data was confirmed with Kolmogorov–Smirov test and equal variance with Levene’s test. Statistical significance was accepted when *p* < 0.05.

## Results

### CHF-induced neuroinflammation and macrophage expansion in the SG

**Fig. 1:**
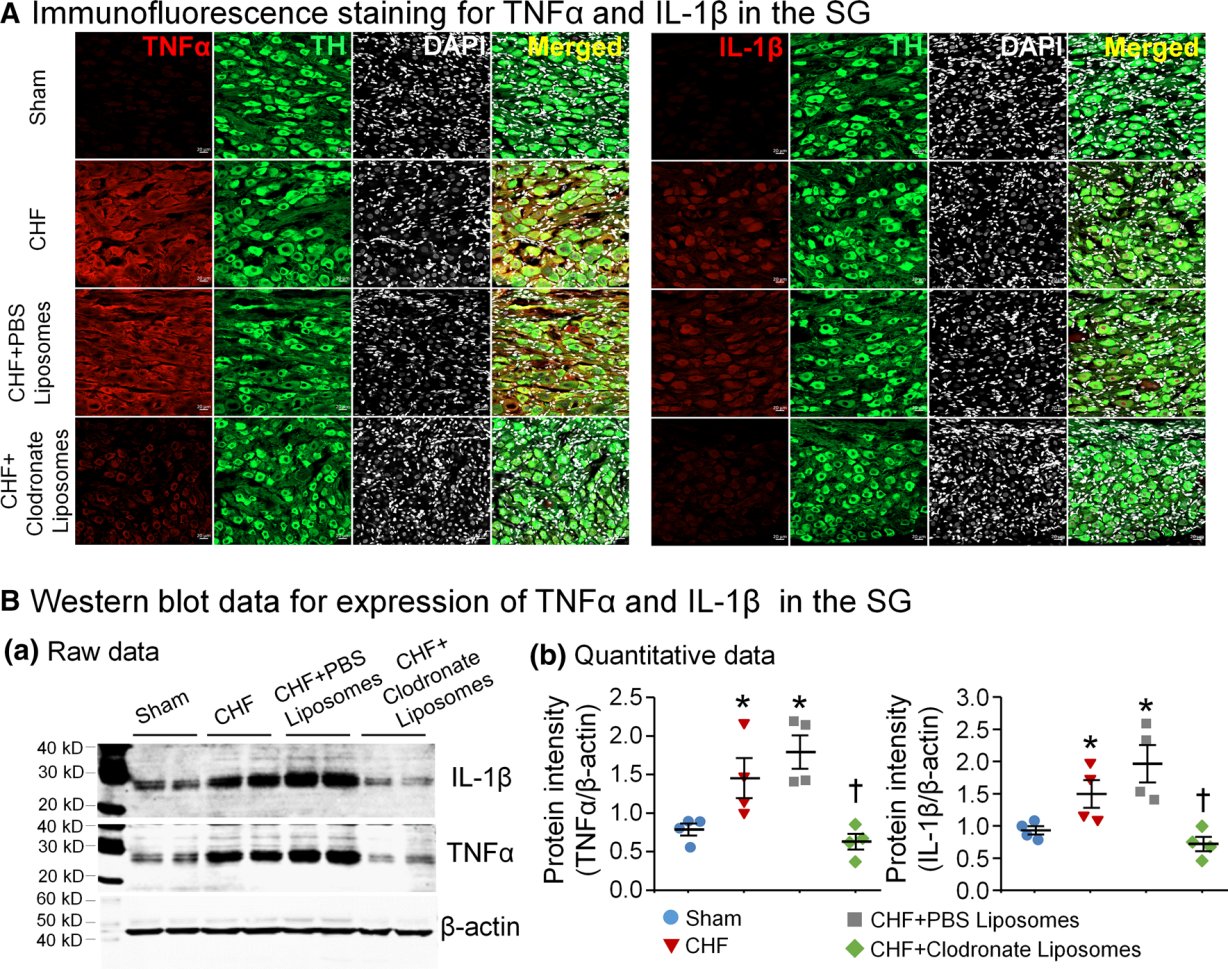
Macrophage depletion in SGs attenuated the neuroninflammation in CHF. **A** Representative images of immunofluorescence staining showing increased levels of proinflammatory cytokines including TNFα (left panel) and IL-1β (right panel) in SGs from CHF rat, compared to sham rat. Microinjection of clodronate liposomes reduced CHF-elevated TNFα and IL-1β levels in SGs. *DAPI* nuclear marker, *TH* tyrosine hydroxylase (an adrenergic neuronal marker). **B** Representative images (**a**) and quantitative data (**b**) showed expression of TNFα and IL-1β proteins in SGs from all groups, measured by Western blot analysis. Macrophage depletion in SGs markedly decreased expression of TNFα and IL-1β in CHF rats. *n* = 4 measurements from 8 rats per group. Statistical significance was determined by one-way ANOVA with post-hoc Bonferroni test. Data are means ± SEM. *p < 0.05 vs. sham; ^†^*p* < 0.05 vs. CHF

We first used a cytokine protein array to screen 19 inflammatory cytokines in SGs from sham and CHF rats (Supplemental Fig. 2a). The data demonstrated that nine cytokines were detectable, and only proinflammatory cytokines including TNFα and IL-1β were elevated in SGs from CHF rats, compared to sham rats. However, there were no significant differences in anti-inflammatory cytokine (IL-10) and other proinflammatory cytokines including cytokine-induced neutrophil chemoattractants (CICN), granulocyte–macrophage colony-stimulating factor (GM-CSF), IL-1α, lipopolysaccharide-induced CXC chemokine (LIX), and vascular endothelial growth factor (VEGF) between sham and CHF rats (Supplemental Fig. 2b, c). The data from immunofluorescence staining (Fig. 1A) and Western blot (Fig. 1B) further confirmed that TNFα and IL-1β levels in SGs were significantly higher in CHF rats than that in sham rats.

**Fig. 2:**
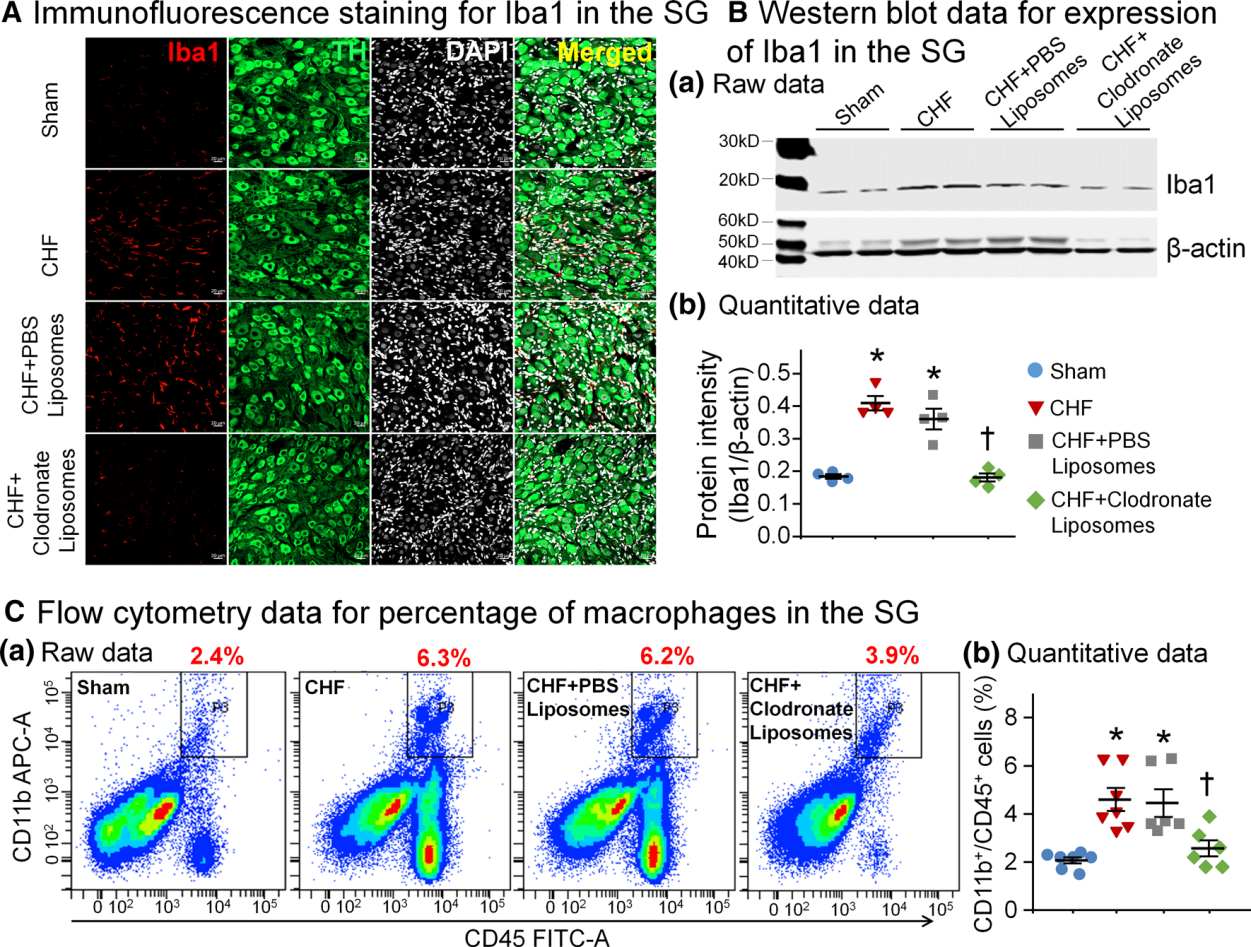
Microinjection of clodronate liposomes into SGs reduced CHF-elevated macrophage activation in SGs. **A** Raw images of immunofluorescence staining showing levels of macrophage activation in SGs from all groups. *Iba1* ionized calcium-binding adaptor molecule 1 protein (a protein marker for macrophage activation), *DAPI* nuclear marker, *TH* tyrosine hydroxylase (an adrenergic neuronal marker). **B** Representative images (**a**) and quantitative data (**b**) of Western blot analysis showing CHF-increased expression of Iba1 protein was reduced by clodronate liposomes. *n* = 4 measurements from 8 rats per group. **C** Representative images (**a**) and quantitative data (**b**) for flow cytometry analysis showing macrophage expansion in SGs from all groups. CHF-increased percentage of macrophages (defined as CD45^+^/CD11b^+^ cells) in SGs was reduced after local treatment with clodronate liposomes. *n* = 6–7 rats per group. Statistical significance was determined by one-way ANOVA with post-hoc Bonferroni test. Data are means ± SEM. *p < 0.05 vs. sham; ^†^*p* < 0.05 vs. CHF

Considering that proinflammatory cytokines are produced predominantly by macrophage activation [14], we then tested if CHF-increased proinflammatory cytokine levels are attributed to macrophage activation in SGs. Iba1 is a microglial/macrophage-specific calcium-binding protein and it is involved in the membrane phagocytosis in microglial/macrophage [31, 56]. Considering that it is widely used as a microglial/macrophage marker in mammalian models due to its specific upregulation during the activation of these cells [44, 51], we used the expression of Iba1 protein to determine macrophage activation in SGs. The data about the expression of Iba1 protein showed that macrophages in SGs were markedly activated in CHF rats, compared to age-matched sham rats (Fig. 2A, B). Using Flow cytometry, our data demonstrated that the percentage of macrophages (defined as CD45^+^/CD11b^+^ cells) in SGs was markedly higher in CHF rats than that in sham rats (4.60 ± 0.47% in CHF rats vs. 2.07 ± 0.13% in sham rats, *p* < 0.05, Fig. 2C).

### In vivo depletion of macrophages reduced macrophage expansion and neuroinflammation in SGs

Macrophage expansion and neuroinflammation were measured at 7 days after clodronate liposomes (2 µl/side, 20 mg/ml, an agent for deletion of macrophages) or PBS liposomes (control) were microinjected into bilateral SGs of CHF rats. The immunofluorescence staining (Fig. 2A) and Western blot (Fig. 2B) data demonstrated that clodronate liposomes significantly reduced the CHF-induced macrophage activation. Additionally, data from flow cytometry further confirmed that macrophage expansion in SGs was reduced by clodronate liposomes, as evidenced by the reduction in the percentage of macrophages in CHF plus clodronate liposomes group (Fig. 2C). Clodronate liposomes also attenuated CHF-elevated TNFα and IL-1β levels in SGs (Fig. 1A and B). Treatment with PBS liposomes had no effect on CHF-elevated macrophage activation and expansion (Fig. 2), as well as proinflammatory cytokine levels in SGs (Fig. 1).

### Macrophage depletion in SGs reduced CHF-increased N-type Ca^2+^ currents and excitability of CSP neurons

**Fig. 3:**
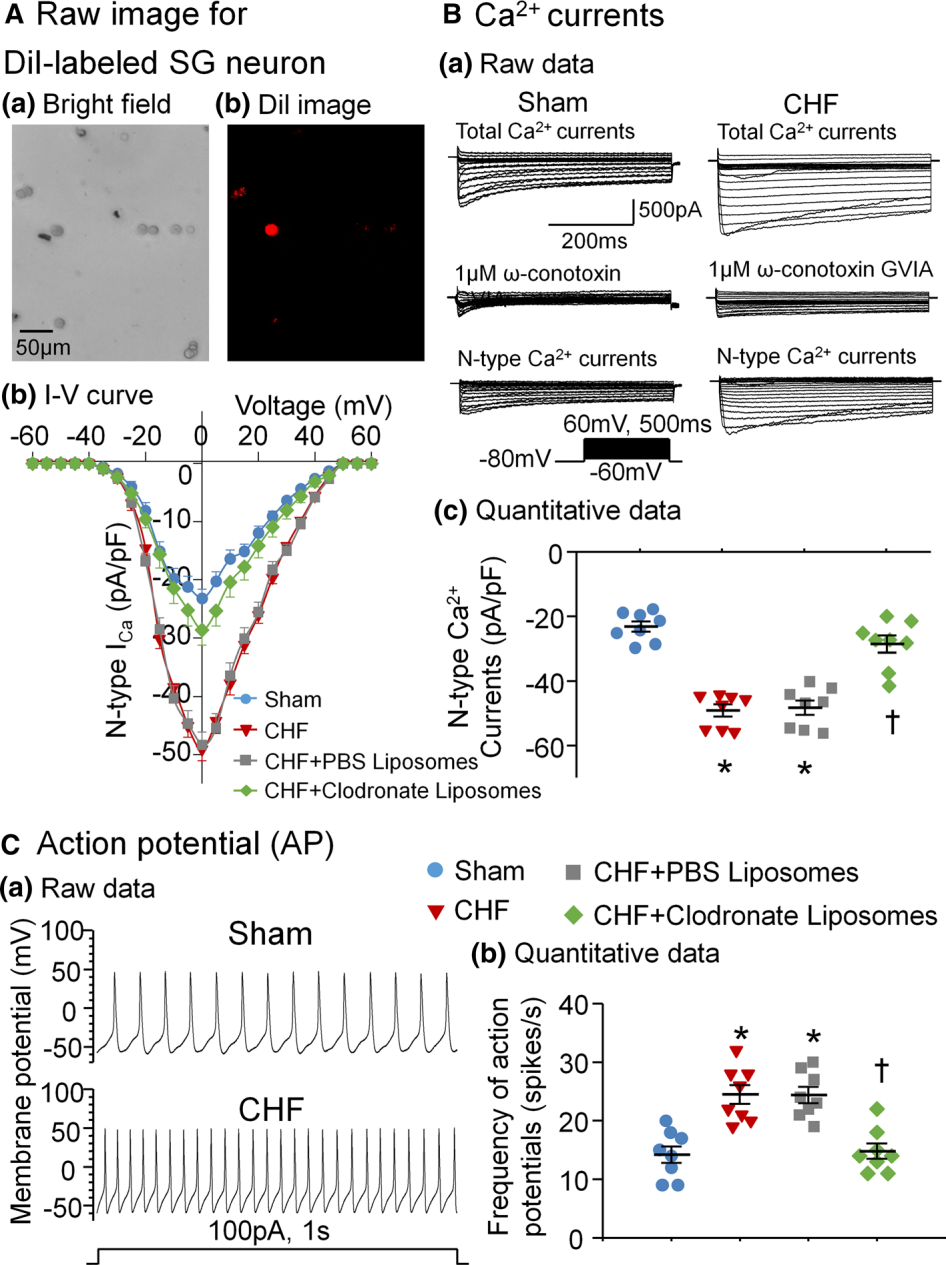
Macrophage depletion in SGs reduced CHF-increased N-type Ca^2+^ currents and excitability of CSP neurons. **A** Bright field monochrome image of SG neurons (**a**) and DiI (red color)-labeled SG neurons (i.e. CSP neurons, **b**). **B** Original recording of Ca^2+^ currents (**a**), current–voltage (I–V) curve of N-type Ca^2+^ currents (**b**), and quantitative data of N-type Ca^2+^ currents elicited by 500-ms test pulse at 0 mV from holding potential of − 80 mV (**c**) in CSP neurons from all groups. **C** Original recording of action potentials (APs, **a**) and quantitative data for frequency of APs (**b**) in CSP neurons from all groups. In vivo microinjection of clodronate liposomes into SGs attenuated CHF-increased N-type Ca^2+^ currents and excitability of CSP neurons. *n* = 8 neurons from 6 rats per group. Statistical significance was determined by one-way ANOVA with post-hoc Bonferroni test. Data are means ± SEM. *p < 0.05 vs. sham; ^†^*p* < 0.05 vs. CHF

Using the whole-cell patch-clamp technique, we recorded N-type Ca^2+^ currents and APs in DiI-labeled SG neurons (i.e. CSP neurons, Fig. 3A). N-type Ca^2+^ currents were obtained by subtracting Ca^2+^ currents under treatment of ω-conotoxin GVIA (a specific N-type Ca^2+^ channel blocker) from total Ca^2+^ currents (Fig. 3Ba). Compared to sham rats, total Ca^2+^ currents (Supplemental Fig. 3a), N-type Ca^2+^ currents (Fig. 3B), and frequency of APs (a parameter of the neuron excitability, Fig. 3c) in CSP neurons were markedly increased in CHF rats, which is consistent with the results from our previous study [58]. Treatment with clodronate liposomes, instead of PBS liposomes, in SGs normalized CHF-increased total Ca^2+^ currents (Supplemental Fig. 3a), N-type Ca^2+^ currents (Fig. 3Bc), and frequency of APs (Fig. 3C) towards the level seen in sham rats. Additionally, there were no significant differences in other types (including L-type, P/Q-type, and R-type) of Ca^2+^ currents, cell membrane capacitance, input resistance, and resting membrane potential among groups (Supplemental Fig. 3b–e).

### Macrophage depletion in SGs attenuated cardiac sympathetic overactivation in both conscious and anesthetized CHF rats

**Fig. 4:**
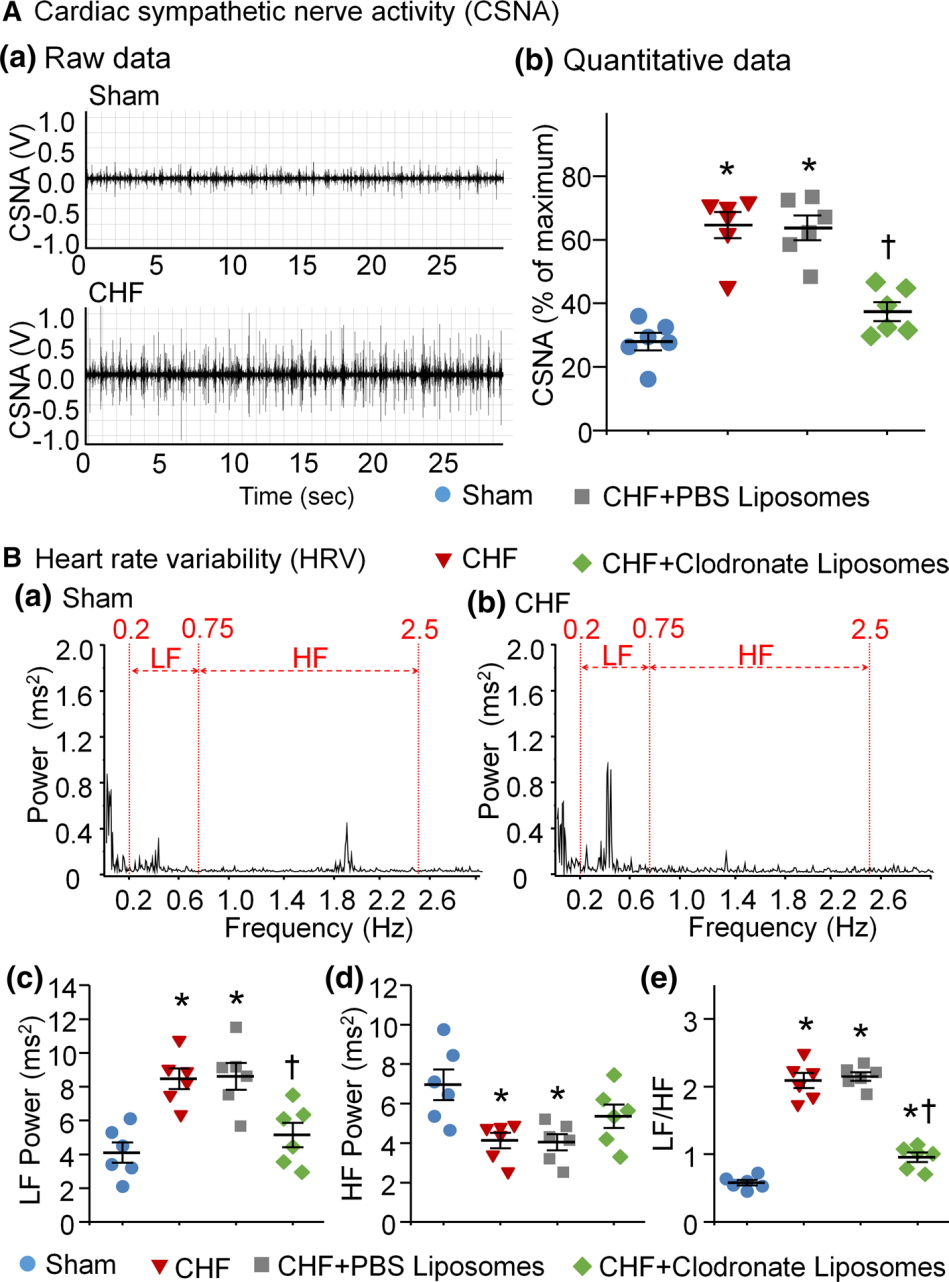
Macrophage depletion in SGs attenuated CHF-induced cardiac sympathetic overactivation in both conscious and anesthetized CHF rats. **A** Representative tracings (**a**) and quantitative data (**b**) for CSNA recorded in all groups of anesthetized rats. Microinjection of clodronate liposomes into SGs markedly reduced CSNA in CHF rats. **B** Representative (**a**–**b**) and quantitative (**c**–**e**) data of HRV analyzed from 24-h ECG recording in conscious rats. Spectral power was quantified for LF from 0.2–0.75 Hz and HF from 0.75–2.5 Hz. Clodronate liposomes attenuated CHF-enhanced LF and LF/HF ratio. LF and LF/HF ratio (indexes of cardiac sympathetic activation) were markedly reduced by clodronate liposomes treated in SGs in CHF. *n* = 6 rats per group. Statistical significance was determined by one-way ANOVA with post-hoc Bonferroni test. Data are means ± SEM. *p < 0.05 vs. sham; ^†^*p* < 0.05 vs. CHF

To assess the effects of macrophage depletion on cardiac sympathetic excitation in CHF rats, we examined the CSNA and LF/HF ratio of HRV spectral analysis, two indexes of cardiac sympathetic activation. In vivo microinjection of clodronate liposomes into SGs significantly decreased the CSNA in anesthetized CHF rats (Fig. 4A) and the LF and LF/HF ratio in conscious CHF rats (Fig. 4Bc and Be). However, clodronate liposomes did not affect HF (an index of cardiac parasympathetic oscillation) in conscious CHF rats (Fig. 4Bd). Additionally, PBS liposomes had no effects on CSNA, LF, HF, and LF/HF ratio in CHF rats (Fig. 4A and B).

### Improved heterogeneity of ventricular electrical activity after macrophage depletion in SGs in CHF

Growing evidence demonstrates that heterogeneity of ventricular electrical activities including QT and QTc intervals, QT and QTc dispersions, and Tpe interval plays an important role in ventricular arrhythmogenesis [2]. Like our previous study [58], the data from 24-h ECG recording in conscious rats showed that CHF significantly prolonged QT and QTc intervals, increased QT and QTc dispersions, and lengthened Tpe interval (Supplemental Fig. 4). Treatment with clodronate liposomes in SGs markedly shortened CHF-caused prolongation of QT and QTc intervals, increases in QT and QTc dispersions, and elongation of Tpe interval (Supplemental Fig. 4). However, treatment with PBS liposomes in SGs failed to restore CHF-increased heterogeneity of ventricular electrical activity (Supplemental Fig. 4).

### In vivo macrophage depletion in SGs attenuated ventricular arrhythmias in both conscious and anesthetized CHF rats

**Fig. 5:**
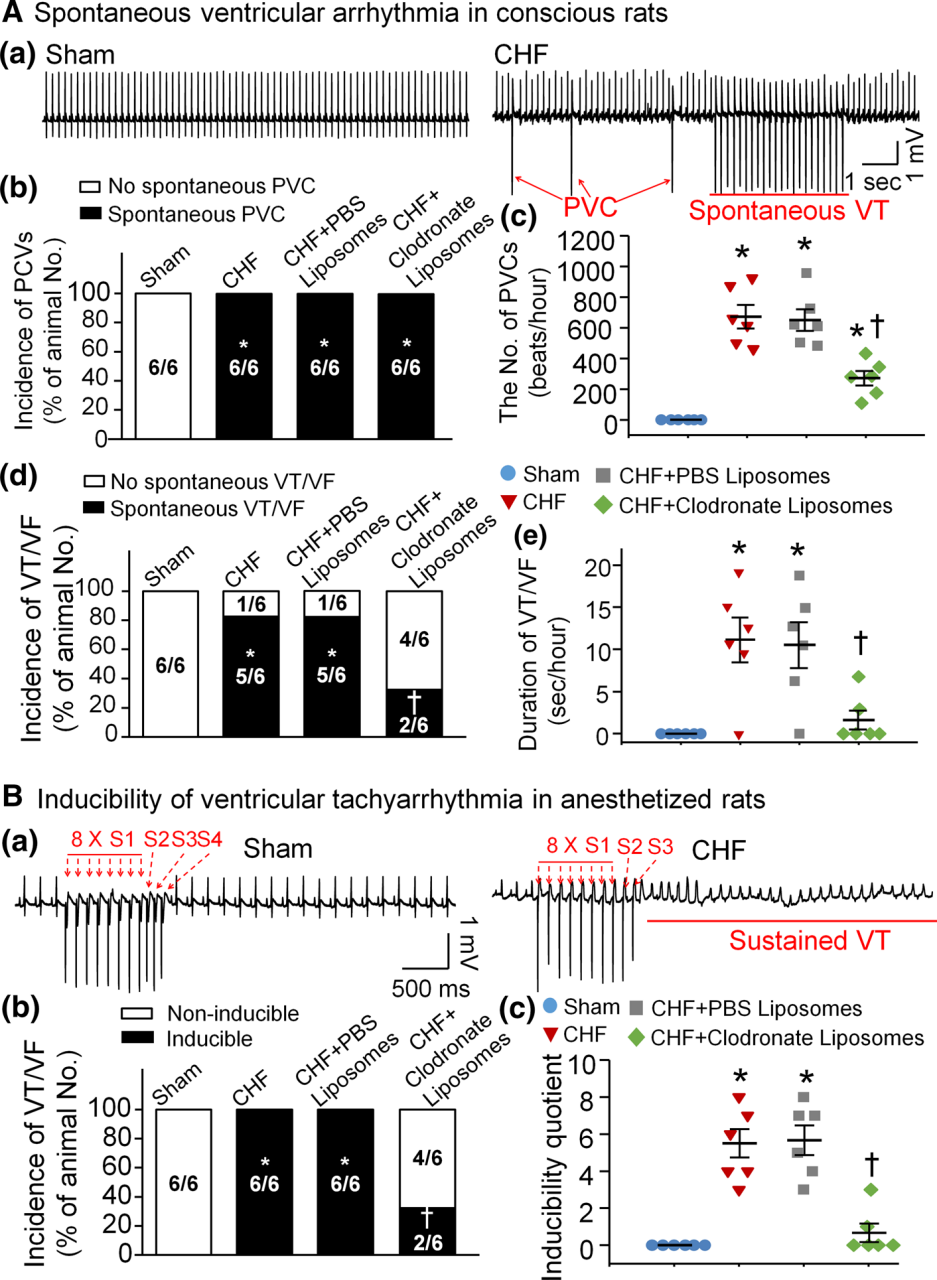
Macrophage depletion in SGs alleviated ventricular arrhythmias in both conscious and anesthetized CHF rats. **A** Raw ECG recordings for PVCs and VT/VF in conscious sham and CHF rats (**a**), and mean data for incidence of PVCs (**b**), the number of PVCs (**c**), incidence of VT/VF (**d**), and cumulative duration of VT/VF (**e**) in all groups of conscious rats. Treatment with clodronate liposomes in SGs significantly reduced the number of PVCs, incidence of VT/VF, and cumulative duration of VT/VF in CHF rats. **B** Raw data for PES-evoked VT/VF in anesthetized sham and CHF rats (**a**), and mean data for incidence (**b**) and inducibility quotient (**c**) of PES-evoked VT/VF in all groups of anesthetized rats. Macrophage depletion with clodronate liposomes in SGs markedly decreased incidence and inducibility quotient of PES-evoked VT/VF in anesthetized CHF rats. *n* = 6 rats per group. Statistical significance was determined by a Chi-Square test for data presented in panel Ab, Ad, and Bb. Statistical significance was determined by one-way ANOVA with post-hoc Bonferroni test for data presented in panel Ac, Ae, and Bc. Data are means ± SEM. *P < 0.05 vs. sham; ^†^*p* < 0.05 vs. CHF

In conscious rats, spontaneous ventricular arrhythmias were monitored and analyzed by 24-h radiotelemetry ECG recording (Fig. 5A). In sham rats, PVCs and VT/VF were not detected. In CHF rats, 100% (6/6) and 83.3% (5/6) of the animals had PVCs and VT/VF, respectively. The number of PVCs and cumulative duration of VT/VF were significantly increased in CHF rats, compared to sham rats (Fig. 5Ac and Ae). Microinjection of clodronate liposomes in SGs markedly reduced the incidence and cumulative duration of VT/VF and the number of PVCs, but it had no effect on the incidence of PVCs (Fig. 5A).

Besides spontaneous ventricular arrhythmias in conscious rats, PES-triggered inducibility of ventricular arrhythmias (including the incidence of VT/VF and inducibility quotient) was tested in anesthetized rats to further clarify the anti-arrhythmic effect of macrophage depletion in CHF (Fig. 5B). In sham rats, PES did not trigger the occurrence of VT/VF, and the inducibility quotient was zero. In CHF rats, PES induced VT/VF with a high incidence (100%) and inducibility quotient (5.33 ± 0.80), compared to sham rats. Treatment with clodronate liposomes in SGs markedly decreased the incidence of VT/VF (33.3%) and inducibility quotient (0.67 ± 0.49) in CHF rats (Fig. 5B). However, treatment with PBS liposomes in SGs had no effect on ventricular arrhythmias in both conscious (Fig. 5A) and anesthetized CHF rats (Fig. 5B).

### Macrophage depletion in SGs failed to improve the performance of the failing heart

**Fig. 6:**
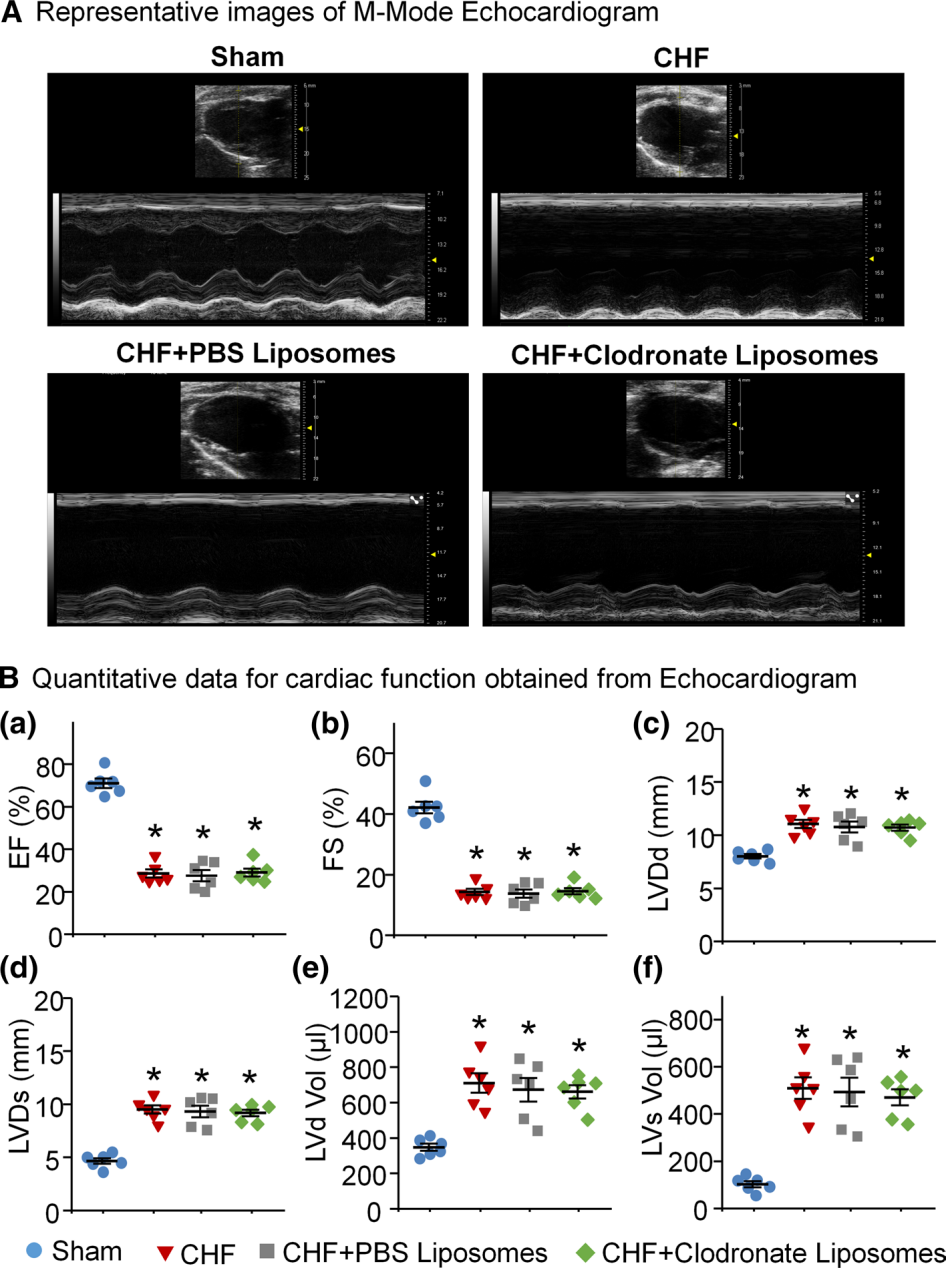
Macrophage depletion in SGs failed to improve cardiac performance in CHF. **A** Representative B-model (upper) and M-mode (bottom) echocardiographic images in left ventricles from Sham, CHF, CHF + PBS Liposomes, and CHF + Clodronate Liposomes rat. **B** Quantitative data EF (**a**), FS (**b**), LVDd (**c**), LVDs (**d**), LVd Vol (**e**), and LVs Vol (**f**) calculated from M-mode images of parasternal long axis view in all groups. *n* = 6 rats per group. Statistical significance was determined by one-way ANOVA with post-hoc Bonferroni test. Data are means ± SEM; *p < 0.05 vs. sham

The data from echocardiography demonstrated that cardiac performance was markedly reduced in CHF rats, compared to sham rats (Fig. 6). In vivo treatment with clodronate liposomes in SGs failed to improve CHF-impaired cardiac contractile function, including EF, FS, LVDd, LVDs, LVd Vol, and LVs Vol (Fig. 6B). Additionally, hemodynamic and morphological data demonstrated that CHF rats presented a huge infarct size in the left ventricle and left ventricular contractile dysfunction (Table 1). Treatment with clodronate liposomes in SGs had no effect on hemodynamic and morphological parameters in CHF rats (Table 1). There were no significant differences in body weight, mean blood pressure (MBP), and heart rate (HR) among groups (Table 1).

## Discussion

### Major findings

For the first time, this study provided direct evidence showing the contribution of macrophage activation and its consequence of neuroinflammation in SGs to cardiac sympathetic overactivation and ventricular arrhythmogenesis in the CHF state. As an agent for macrophage deletion, in vivo microinjection of clodronate liposomes into SGs significantly decreased the levels of TNFα and IL-1β in SGs from CHF rats. CHF-increased N-type Ca^2+^ currents and excitability of CSP neurons were also attenuated by local treatment with clodronate liposomes. Additionally, clodronate liposomes not only blunted cardiac sympathetic overactivation, but also decreased the occurrence of ventricular arrhythmias and heterogeneity of ventricular electrical activities in anesthetized and conscious CHF rats. However, clodronate liposomes failed to improve CHF-induced cardiac contractile dysfunction. Using the strategy of macrophage depletion, our data confirm a direct, positive correlation between neuroninflammation in SGs and ventricular arrhythmogenesis in CHF. These data suggest that macrophage depletion or anti-inflammation in SGs could be a potential therapeutic strategy to suppress lethal ventricular arrhythmias and reduce mortality in the CHF state.

### Macrophage activation and neuroinflammation in the SG

MI induces time-dependent changes in the neuroendocrine and expression of various genes [13]. Systemic inflammation after MI indeed serves as the primer for the subsequent occurrence of neuroinflammation in CHF [48]. In the present study, macrophage activation in SGs was confirmed to contribute to the neuroinflammation in the CHF state. From our current study, however, it is unclear why macrophages were over-activated in SGs. Additionally, a combination of CD11b and CD45 labeling is widely used to distinguish microglia from macrophages in many studies [4, 10, 12, 45], which was also employed by our present study to identify macrophages when the cells were double positive with CD45/CD11b. However, it is important to note that CD11b is not only expressed on macrophages, but also expressed on the surface of other leukocytes including monocytes, neutrophils, granulocytes, and natural killer cells [7], and these leukocytes are also CD45^+^ cells [26, 27]. Therefore, the above issues including the macrophage overactivation and specific macrophage markers are required to be further identified by future studies.

It has been reported that proinflammatory cytokines such as TNFα and IL-1 are elevated in SGs from patients with a variety of cardiomyopathies and correlated with prognosis and severity of CHF [11, 16]. However, the direct contribution of neuroinflammation (defined as sustained macrophage activation and production of proinflammatory cytokines) [30] to autonomic dysfunction is not understood. Recently, many studies have greatly recognized the role of neuroinflammation in cardiac sympathetic overactivation and arrhythmogenesis, especially the involvement of inflammatory infiltration in patients with severe ventricular arrhythmias [1, 40]. Similarly, our current study found that the levels of proinflammatory cytokines including TNFα and IL-1β in SGs were markedly increased in CHF rats. Although the main source of TNFα and IL-1β in SGs was not clarified, several possible sources contributing to CHF-elevated the levels of proinflammatory cytokines in SGs are considered below. First, activated macrophages that originally resided in SGs (converted from microglia) and recruited from circulation (derived from circulating monocytes) are thought to be the key source of proinflammatory cytokines in local tissues [22, 54]. It is supported by our current study that macrophages were markedly activated in SGs from CHF rats (Fig. 2). Second, proinflammatory cytokines recruited from circulation (endocrine fashion) [60] possibly serve as one source for CHF-increased levels of TNFα and IL-1β in SGs. Third, based on the projection of CSP neurons located in SGs to the heart, proinflammatory cytokines retrograded from cardiac myocytes through axons might be another source for CHF-increased levels of proinflammatory cytokines in SGs. One previous study has demonstrated that myocardium-retrograded cytokines could be a key factor to induce CSP neuronal remodeling in SGs [37]. Finally, considering the fact that TNFα itself can promote an M1-like fate in macrophages to trigger the secretion of TNFα and IL-1β from macrophages [8], the interaction among these sources of proinflammatory cytokines could form a vicious circle to elevate local levels of TNFα and IL-1β in SGs from CHF rats.

### Proinflammatory cytokines promote cardiac sympathetic overactivation through affecting N-type Ca^2+^ channels in CSP neurons in CHF

Growing evidence has confirmed that VDCCs contribute to cell excitability, intracellular Ca^2+^ level, and neurotransmitter release in the central and peripheral nervous systems. Thus far, four types (L, N, P/Q, and R) of VDCCs have been reported to be located in CSP neurons. Using human heart atrium, Molderings et al. found that N-type, but not L- and P/Q-type, Ca^2+^ channels regulate neurotransmitter release from cardiac sympathetic nerve terminals [32]. Our previous study demonstrated that about 60–70% of whole Ca^2+^ currents are produced by N-type Ca^2+^ channels in CPS neurons, and only N-type Ca^2+^ currents and cell excitability of CSP neurons are increased in CHF rats [50]. More importantly, our recent study used in vivo transfection of Ca_v_2.2-α shRNA into CSP neurons to normalize CHF-increased N-type Ca^2+^ currents and cardiac sympathetic activation [58]. Therefore, we believe that CHF-increased N-type Ca^2+^ currents in CSP neurons could be a key factor for cardiac sympathetic overactivation.

However, the mechanisms responsible for CHF-increased N-type Ca^2+^ currents in CSP neurons are not understood. The data from RNA sequence analysis demonstrated a significant induction of inflammatory signal in both SGs and dorsal root ganglia following pig chronic MI [13]. Using single-cell patch-clamp recording, Wilkinson et al. found that proinflammatory cytokines such as TNFα and IL-1β activated VDCCs in isolated rat vascular smooth muscle cells [53]. Additionally, neuroinflammation can boost sympathetic activation in both clinical and animal experimental studies [1, 33]. Our present study demonstrates that CHF induces the elevation of proinflammatory cytokines (TNFα and IL-1β) mainly derived from activated macrophages in SGs (Figs. 1, 2), which is accompanied by increases in N-type Ca^2+^ currents of CSP neurons (Fig. 3) and cardiac sympathetic overactivation (Fig. 4). In particular, local in vivo microinjection of clodronate liposomes into SGs simultaneously inhibits CHF-induced elevation of TNFα and IL-1β, increases in N-type Ca^2+^ currents, and cardiac sympathetic overactivation. These data provide direct evidence to clarify the involvement of proinflammatory cytokines of CSP neurons in cardiac sympathetic overactivation through affecting the activation of N-type Ca^2+^ channels in the CHF state. However, our current study cannot answer how proinflammatory cytokines modulate N-type Ca^2+^ channels, which requires further investigation.

### Local depletion of macrophages with clodronate liposomes in SGs is a potential strategy for suppression of ventricular arrhythmias

Since proinflammatory cytokines are predominantly released from activated macrophages [14], depleting macrophages allows us to test if proinflammatory cytokines contribute to CHF-induced cardiac sympathetic overactivation and ventricular arrhythmias. Systemic injection of clodronate liposomes is perhaps the easiest way to deplete macrophages and has been used for many years [19]. This method relies upon phagocytosis of the clodronate liposomes by macrophages, resulting in the intracellular release of clodronate and subsequent macrophage apoptosis [42]. Given that systemic injection of clodronate liposomes may eliminate macrophages regardless of origin throughout the body [19], an ideal strategy for achieving organ-specific macrophage depletion is local microinjection of clodronate liposomes. This approach has been applied to eliminate perivascular macrophages specifically in the brain [55]. In the present study, therefore, clodronate liposomes were microinjected into SGs for specifically depleting macrophages in SGs. This treatment with clodronate liposomes markedly reduced CHF-elevated macrophage expansion (Fig. 2) and inhibited CHF-increased proinflammatory cytokines including TNFα and IL-1β in SGs (Fig. 1). These data suggest that local depletion of macrophages is an effective therapeutic strategy for anti-inflammation in the SG in CHF.

Using local depletion of macrophages, our current study directly tested whether macrophage activation in SGs aggravates CHF-induced ventricular arrhythmias. Considering the association of systemic inflammation with CHF [48], it is possible that macrophage expansion in other organs such as the brain and heart might be involved in CHF-induced sympathetic overactivation. By macrophage depletion in the central nervous system, Yu et al. reported that proinflammatory cytokines stimulate central sympathetic excitation through activating perivascular macrophages in paraventricular nucleus after acute MI [55]. Although systemic elimination of macrophage has also been reported to suppress cardiac sympathetic hyperinnervation following acute MI [52], the exact pathological contribution of systemic inflammation to cardiac sympathetic excitation and ventricular arrhythmias in the CHF state should be further addressed.

### Perspectives and study limitations

Clinical studies reported that inflammatory infiltration in SGs was significantly increased in patients with severe arrhythmias in the late stage of cardiomyopathy or long QT syndrome [1, 40]. Our previous study demonstrated that fatal ventricular arrhythmias mainly occur in the early stage (1–3 weeks post- MI) and late stage (12–14 weeks post-MI) of CHF rats [59]. Our present study strongly supports the correlation between neuroinflammation in SGs and severe ventricular arrhythmias in the late stage of some heart diseases demonstrated by clinical studies [1, 40]. Our study also provides a potential therapeutic strategy that macrophage depletion or anti-inflammation in the late stage of CHF would effectively suppress fatal ventricular arrhythmias through inhibition of macrophage expansion and neuroninflammation in SGs. However, there are some limitations in the current study. First, clodronate liposomes failed to improve the contractile performance of the failing heart in the current study. One reasonable explanation is that clodronate liposomes could not reduce the infarct size and cardiac hypertrophy already formed during the progression of HF when this drug was applied in the late stage of CHF (12 weeks post-MI). Accordingly, we believe that instead of focusing treatment on the developed failing heart, effective pharmacological interventions in proarrhythmic factors such as macrophage expansion and neuroninflammation might be more appropriate in the management of cardiac sympathetic overactivation and ventricular arrhythmias in CHF. Additionally, further studies are needed to clarify whether macrophage depletion or anti-inflammation in the early stage of CHF is beneficial to the failing heart for improvement of cardiac contractile function and suppression of ventricular arrhythmias. Moreover, while treatment with clodronate liposomes in SGs successfully attenuated the CHF-induced cardiac sympathetic overactivation and ventricular arrhythmias, the distinct roles of resident and nonresident macrophages in CHF-induced cardiac sympathetic overactivation and ventricular arrhythmias require further in-depth analyses. Second, although inflammatory infiltration in SGs was measured by immunofluorescence staining, western blot, and flow cytometry in the present study, the histological measurement would provide further morphological support to the increase of macrophages in SGs. Therefore, the histological measurement will be done in future studies as the direct evidence for macrophage infiltration in SGs. Finally, using the dog model of acute myocardial ischemia, Heusch et.al demonstrated that myocardial ischemia increases cardiac sympathetic activity by activating spinal cardiocardiac sympathetic reflex [17]. Although our current study demonstrated that CHF-induced cardiac sympathetic overactivation at least partially due to neuroinflammation-activated N-type Ca^2+^ channels in CSP neurons, this result cannot rule out the involvement of a vicious cycle (positive feedback) between cardiac sympathetic overactivation and severe myocardial ischemia. Further studies are required to explore whether spinal reflex contributes to cardiac sympathetic overactivation during the progression of MI-induced CHF.

## Conclusion

**Fig. 7:**
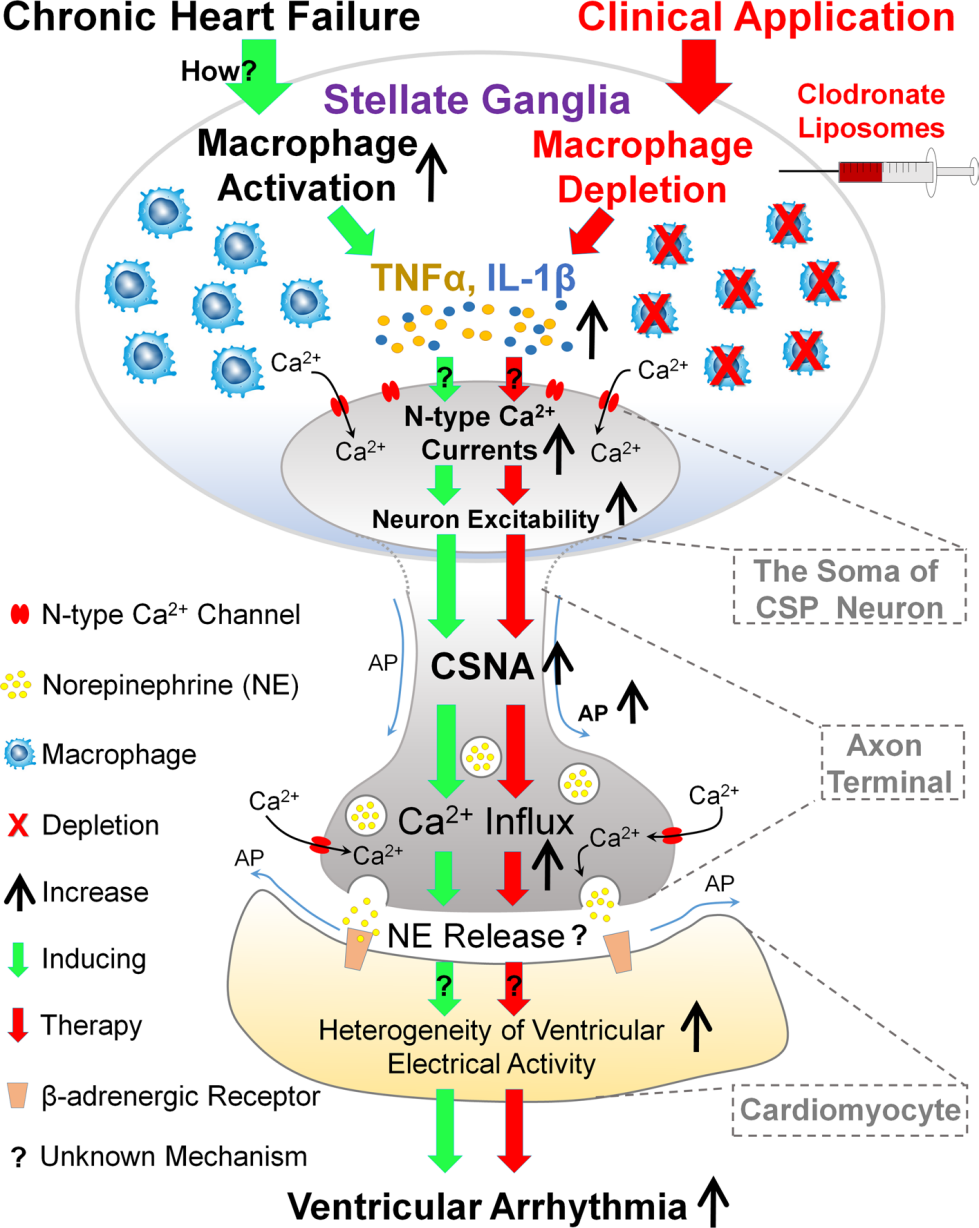
Contribution of macrophage activation to ventricular arrhythmias in CHF and potential clinical applications. Macrophage activation in SGs aggravates cardiac sympathetic overactivation and ventricular arrhythmias via inducing proinflammatory cytokine-elevated N-type Ca^2+^ currents and excitability of CSP neurons. Local macrophage depletion with clodronate liposomes in SGs attenuates neuroinflammation-activated N-type Ca^2+^ channels in CSP neurons, which subsequently reduces Ca^2+^ influx and CSNA in CHF. Finally, lethal ventricular arrhythmia is suppressed by attenuating cardiac sympathetic overactivation-increased heterogeneity of ventricular electrical activities in CHF. Macrophage depletion and anti-inflammatory treatment in SGs could be a potential therapeutic strategy to suppress cardiac sympathetic overactivation and ventricular arrhythmias in the CHF state

In summary, our present study demonstrates that macrophage depletion with clodronate liposomes in SGs attenuates overactivation of N-type Ca^2+^ channels and excitability in CSP neurons through reducing the levels of proinflammatory cytokines in SGs, which subsequently decreases cardiac sympathetic overactivation and ventricular arrhythmias in CHF rats (Fig. 7). From these results, we believe that future studies will be designed to access the therapeutic potential of macrophage depletion or anti-inflammatory treatment in SGs on-top-of β-blockers which are widely used in patients with CHF.

## Supplementary Information

Below is the link to the electronic supplementary material.

Supplementary file1 (DOCX 605 kb)

## Data Availability

The datasets generated and/or analyzed during the current study are not publicly available due to confidentiality reasons but are available from the corresponding author on reasonable request.
