# Supplementary material for: Macrophage depletion in stellate ganglia alleviates cardiac sympathetic overactivation and ventricular arrhythmogenesis by attenuating neuroinflammation in heart failure
Source: Basic Res Cardiol. 2021 Apr 21;116(1):28. doi: 10.1007/s00395-021-00871-x (PMC8060235; doi:10.1007/s00395-021-00871-x)
Supplement: Supplementary file 1 — Supplementary file1 (DOCX 605 kb) [file 395_2021_871_MOESM1_ESM.docx]

SUPPLEMENTAL MATERIALS TO

**Macrophage depletion in stellate ganglia alleviates cardiac sympathetic overactivation and ventricular arrhythmogenesis by attenuating neuroinflammation in heart failure**

Dongze Zhang, MD,^1†^* Wenfeng Hu, PhD,^1†^ Huiyin Tu, PhD,^1†^ Bryan T. Hackfort, PhD,^2^ Bin Duan, PhD,^3^ Wanfen Xiong, MD, PhD,^4^ Michael C. Wadman, MD,^1^ Yu-Long Li, MD, PhD^1,2^*

^1^Department of Emergency Medicine, University of Nebraska Medical Center, Omaha, NE 68198, USA; ^2^Department of Cellular & Integrative Physiology, University of Nebraska Medical Center, Omaha, NE 68198, USA; ^3^Mary & Dick Holland Regenerative Medicine Program, Division of Cardiology, Department of Internal Medicine, University of Nebraska Medical Center, Omaha, NE 68198, USA; ^4^Department of Internal Medicine, University of Nebraska Medical Center, Omaha, NE 68198, USA

**^†^These authors contributed equally to the study.**

***Corresponding authors:**

**Yu-Long Li, MD, PhD.** Department of Emergency Medicine, University of Nebraska Medical Center, 985850 Nebraska Medical Center, Omaha, NE 68198-5850, USA

Tel.: +1-402-559-3016; Fax: +1-402-559-9659; E-mail: [yulongli@unmc.edu](mailto:yulongli@unmc.edu)

**Dongze Zhang, MD.** Department of Emergency Medicine, University of Nebraska Medical Center, 985850 Nebraska Medical Center, Omaha, NE 68198-5850, USA

Tel.: +1-402-559-5922; Fax: +1-402-559-9659; E-mail: [dongze.zhang@unmc.edu](mailto:dongze.zhang@unmc.edu)

**Supplemental Methods**

***Animal model***

All rats were housed two per cage under controlled temperature and humidity and a 12:12-h dark-light cycle, and they were provided water and rat chow ad libitum. CHF rats underwent surgical ligation of the LAD, and sham rats underwent the same surgery without LAD ligation, as previously described [26, 27]. Briefly, under the anesthetized condition (2% isoflurane, Butler Schein Animal Health, Dublin, OH, USA), the left thoracotomy was performed at the 4^th^ intercostal space. In the CHF group, the left coronary artery was ligated with 6-0 silk suture, just below its exit from the aorta, between the pulmonary artery outflow tract and left atrium. Prior to the ligation, 0.05 ml of 1% lidocaine was injected intramuscularly and 3 drops of 1% lidocaine were applied directly to the heart to prevent fatal arrhythmias. The chest and surgical incision were closed, and the terminal experiments were performed at 12-14 weeks after LAD ligation. In the terminal experiment, a Millar pressure transducer was used to further determine left ventricle end-diastolic pressure (LVEDP) and systolic pressure (LVSP). A colorimetric technique was used to measure the infarct size (see detail below). A rat with both LVEDP >15 mmHg and infarct size >30% of the left ventricle was considered as CHF. CHF was confirmed by multiple morphological and hemodynamic parameters (Table 1). 22 rats in CHF groups were excluded from the study, in which 13 rats died within 1 week after surgical ligation of the LAD, 4 rats died within 12 to 14 weeks after surgical ligation of the LAD, and 5 rats were not considered as CHF due to insufficient LVEDP and infarct size.

***In vivo microinjection of clodronate liposomes into SGs***

Under anesthetized condition (2% isoflurane) and mechanical ventilation, the rat was kept in a lateral recumbent position. Bilateral thoracotomy was performed in the second intercostal spaces. After SGs located at the level of cervical vertebrae 7 and anterior to the neck of the first rib was identified, clodronate liposomes (Clophosome, 2 µl, 20mg/ml, CAT: F70101C-AH, FormuMax Scientific Inc, Sunnyvale, CA, USA) or PBS liposomes (CAT: F70101-AH) were microinjected into the bilateral SGs by a glass micropipette connected to a WPI Nanoliter 2000 microinjector. After microinjection, the chest was closed, and the experiments were performed 1 week later.

***Implantation of the ECG telemeter***

Implantation of the ECG telemeter (Millar Instruments, Houston, TX, USA) was performed as described previously [2, 16, 20]. On the day at 11 weeks after LAD ligation (CHF groups) or the same surgery without LAD ligation (sham groups), the rat was anesthetized with 2% isoflurane (Butler Schein Animal Health, Dublin, OH, USA). The skin was shaved and sterilized. After laparotomy was performed at the Linea Alba (abdomen), an ECG transmitter was placed into the abdominal cavity and secured to the abdominal wall at the best position for battery recharging and signal communication. In accordance with the Millar User Manual for ECG recording, the bipolar electrodes were tunneled subcutaneously. The negative electrode was secured in the upper sternal midline and the positive electrode was attached to the underlying tissue near the left side of the xiphoid process. To reduce the electrical noise during the recording, the electrodes were kept together and run alongside one another as far as practical. All incisions were sutured in two layers. ECG recording was performed one week after surgery.

***ECG recording in conscious rats***

One week after implantation of the ECG telemeter, the rat was placed on a SmartPad receiver (Millar Instruments, Houston, TX, USA). For quantification of ventricular arrhythmic events, 24-hour continuous ECG signals were acquired in unrestrained, conscious rats. Real-time ECG signals were digitalized and analyzed by PowerLab 8/30 Data Acquisition System with LabChart 8 software and ECG analysis module (AD Instruments, Colorado Springs, CO, USA). The number of premature ventricular contractions (PVCs) and the cumulative duration of ventricular tachycardia/fibrillation (VT/VF) were manually counted during 24-hour continuous ECG recording. VT was defined as PVCs lasting ≥4 beats. VF was defined as rapid, irregular QRS complexes. QT and corrected QT (QTc) intervals, as well as dispersions, were calculated and averaged from eight 5-minute ECG segments during the 24-hour recording in conscious rats. QTc interval was calculated by Bazett’s formula (QT/$\sqrt{RR}$, where RR is RR interval) [6]. As an index of the spatial dispersion of the ventricular repolarization, QT and QTc dispersions were calculated by equations: QT dispersion = QT_max_ - QT_min_ and QTc dispersion = QTc_max_ - QTc_min_, where QT_max_ and QTc_max_ are the maximum QT interval and the maximum QTc interval; QT_min_ and QTc_min_ are the minimum QT interval and the minimum QTc interval. T-peak to T-end (Tpe) interval, another marker of transmural dispersion of the ventricular repolarization, was calculated and served as an ECG marker of ventricular arrhythmia [1, 23, 24].

***Measurement of HRV in conscious rats***

HRV measurement is a widely used approach for the determination of autonomic function in the conscious state in the clinic. Therefore, HRV measurement served as an alternative method for comparing sympathetic activation in conscious rats in the current study. For quantification of HRV, 24-hour continuous ECG signals were acquired in unrestrained, conscious rats. Real-time ECG signals were digitalized by PowerLab 8/30 Data Acquisition System with LabChart 8 software and HRV analysis module (AD Instruments, Colorado Springs, CO, USA). HRV was analyzed and averaged from eight, 5-minute ECG segments during the 24-hour recording in conscious rats. HRV analysis including low frequency power (LF) from 0.2-0.75Hz, high frequency power (HF) from 0.75-2.5Hz, and LF/HF ratio was performed in the current study [4, 14, 18].

***Labeling of SG neurons***

SG neurons project not only to the heart, but also to other target organs. To explore the relationship between neuroinflammation of CSP neurons and ventricular arrhythmia, we used a transported fluorescent dye (red color DiI) to retrogradely label SG neurons projecting to the cardiac myocardium. Under anesthetized condition (2% isoflurane) and mechanical ventilation, the thoracotomy was performed in the fourth intercostal space. Twenty injections (2 μl DiI for each injection) were made subepicardially into left and right atria and ventricles using a fine-tipped glass micropipette connected to a microinjector (Nanoliter 2000, WPI, Sarasota, FL, USA) [17, 26]. The surgical incision was closed, and isolation of SG neurons for patch-clamp recording in DiI-labeled SG neurons was performed at least 1 week after surgery to allow the dye to diffuse to SG neurons.

***Isolation of SG neurons and whole cell patch-clamp recording for Ca^2+^ currents and action potentials (APs)***

After the thoracotomy was performed under anesthetized condition (800 mg/kg urethane combined with 40 mg/kg α-chloralose, i.p.), bilateral SGs were exposed and removed quickly. SG neurons were isolated by a two-step enzymatic digestion protocol as described previously [22, 26]. Briefly, isolated SGs were placed in ice-cold modified Tyrode’s solution (mM): 140 NaCl, 5 KCl, 10 HEPES, 5 glucose. The SG was then minced into small pieces with microscissors and incubated with a modified Tyrode’s solution containing 0.1% collagenase and 0.1% trypsin for 30 min at 37°C. The tissue was then transferred to a modified Tyrode’s solution containing 0.2% collagenase and 0.5% bovine serum albumin for 30 min of incubation at 37°C. The isolated SG neurons were cultured at 37°C in a humidified atmosphere of 95% air-5% CO_2_ for 4-8 hours before patch-clamp experiments.

Voltage-gated Ca^2+^ currents and APs were recorded only in DiI-labeled SG neurons (i.e. CSP neurons) by the whole-cell patch-clamp technique using Axopatch 200B patch-clamp amplifier (Axon Instruments) [26]. Resistance of the patch pipette was 4-6 MΩ when filled with following solution (in mM): 120 CsCl, 1 CaCl2, 40 HEPES, 11 EGTA, 4 MgATP, 0.3 Tris-GTP, 14 creatine phosphate, and 0.1 leupeptin (pH 7.3; 305 mosM). The extracellular solution consisted of (in mM): 140 TEA-Cl, 5 BaCl2, 1 MgCl2, 10 HEPES, 0.001 TTX, 2 4-AP, and 10 glucose (pH 7.4; 310 mosM). Series resistance of 5-13 MΩ was electronically compensated 30–80%. Junction potential was calculated to be +7.9 mV using pCLAMP 10.2 software, and all values of membrane potential given throughout were corrected using this value. Current traces were sampled at 10 kHz and filtered at 5 kHz. The holding potential was −80 mV and current-voltage (I−V) relationships were elicited by 5-mV step increments to potentials between −60 mV and 60 mV for 500 ms. Peak currents were measured for each test potential, and current density was calculated by dividing peak current by cell membrane capacitance. In patch-clamp experiments, ω-conotoxin GVIA (Alomone Labs), a specific N-type Ca^2+^ channel blocker, was used to block N-type Ca^2+^ channel. Based on the previous study, the concentration of ω-conotoxin GVIA (1 μM) used in the present study is a saturating concentration for inhibiting N-type Ca^2+^ channels [10, 22, 26]. N-type Ca^2+^ currents were obtained by subtracting Ca^2+^ currents under treatment of ω-conotoxin GVIA from total Ca^2+^ currents [22, 26].

In current-clamp experiments, AP was elicited by a current injection of 100 pA, and the frequency of APs was measured in a 1-sec current clamp. The patch pipette solution was composed of (in mM): 105 K-aspartate, 20 KCl, 1 CaCl_2_, 5 MgATP, 10 HEPES, 10 EGTA, and 25 glucose (pH 7.2; 320 mOsm/L). The bath solution was composed of (in mM): 140 NaCl, 5.4 KCl, 0.5 MgCl_2_, 2.5 CaCl_2_, 5.5 HEPES, 11 glucose, and 10 sucrose (pH 7.4; 330 mOsm/L). Junction potential was calculated to be +12.3 mV and membrane potential was corrected using this value. P-clamp 10.2 program (Axon Instruments) was used for data acquisition and analysis. All experiments were performed at room temperature (22–24°C).

***Measurement of inducibility of ventricular tachyarrhythmia in anesthetized rats***

Under anesthetized condition (800 mg/kg urethane combined with 40 mg/kg α-chloralose, i.p.), surface lead-II ECG was recorded using subcutaneous electrodes connected to a biological amplifier (AD Instruments, Colorado Springs, CO, USA). Then, left thoracotomy was performed in the fourth intercostal space. After the heart was exposed, the pericardium was carefully removed. A bipolar platinum stimulating electrode was placed on the right ventricular outflow tract for electrical stimulation [11]. Programmed electrical stimulation (PES) was performed by a programmed electrical stimulator (Digital Pulse Generator 1831; WPI, USA) and an isolator (A320R Isostim Stimulator; WPI, USA). The pulse current output was set to twice the capture threshold and a 2-ms pulse width. To determine the ventricular effective refractory period, a train of eight stimuli (8×S1) at a 120 ms cycle length was applied, followed by an extra-stimulus (S2). Starting at 90 ms, the S1–S2 interval was reduced in steps of 2 ms until the ventricular effective refractory period was identified [7]. Based on the ventricular effective refractory period, a programmed stimulation protocol combined by single (S2), double (S3), or triple extra-stimulus (S4) after a train of eight stimuli (8×S1) was designed to induce ventricular tachyarrhythmia as described previously [9, 11, 20]. The end point of ventricular pacing was induction of ventricular tachyarrhythmia. Ventricular tachyarrhythmia was considered as non-inducible when either PES failed to induce ventricular premature beats or self-terminated ventricular premature beats < 6. Ventricular tachyarrhythmia was considered as non-sustained when it lasted ≤ 15 beats and sustained when it lasted > 15 beats before spontaneously terminating [3, 15].

Inducibility of ventricular tachyarrhythmia was quantified by a quotient of ventricular arrhythmia score as described previously [11, 15]. Zero, non-inducible preparations; 1, non-sustained tachyarrhythmias induced with 3 extra-stimuli; 2, sustained tachyarrhythmias induced with 3 extra-stimuli; 3, non-sustained tachyarrhythmias induced with 2 extra-stimuli; 4, sustained tachyarrhythmias induced with 2 extra-stimuli; 5, non-sustained tachyarrhythmias induced with 1 extra-stimulus; 6, sustained tachyarrhythmias induced with 1 extra-stimulus; 7, tachyarrhythmias induced during a train of 8 stimuli (8×S1) at a basic cycle length of 120 ms; 8, the heart stopped before PES.

***Recording of CSNA*** ***in anesthetized rats***

CSNA was recorded in anesthetized rats (800 mg/kg urethane combined with 40 mg/kg α-chloralose, i.p.), as described previously [25, 26]. Left thoracotomy was performed in the second intercostal space to expose the left SG. Left cardiac sympathetic nerve (CSN) was then dissected distal to the left SG. The CSN was identified by increases in heart rate and blood pressure in response to the sympathetic nerve stimulation. The central cut end of the cardiac sympathetic nerve was placed on a bipolar platinum recording electrode and covered by Kwik-Cast Silicone elastomer (World Precision Instruments). CSNA was recorded using Grass P-55 pre-amplifier (Astro-Med, Inc.) and Chart 8 software (AD Instrument). At the end of the experiment, the maximum CSNA was recorded when the rat was euthanized with an overdose of 3M KCl. Cardiac sympathetic nerve activity was expressed as the percentage of the maximum CSNA.

***Measurement of hemodynamic and morphological parameters***

After the rat was anesthetized (800 mg/kg urethane combined with 40 mg/kg α-chloralose, i.p.), the trachea was cannulated to facilitate mechanical respiration. The animal’s body temperature was maintained at 37°C with an animal temperature controller (ATC 1000; World Precision Instruments, Sarasota, FL, USA). Right femoral vein was cannulated with a polyethylene-50 catheter for the administration of drugs. Left femoral artery was cannulated with a polyethylene-50 catheter for blood pressure and heart rate monitoring. A Millar pressure transducer (SPR 524; size, 3.5-Fr; Millar Instruments, Houston, TX, USA) was slowly inserted into the right carotid artery and carefully advanced to the left ventricle for measurement of LVSP and LVEDP. Hemodynamic data were recorded by PowerLab 8/30 Data Acquisition System with LabChart 8 software (AD Instruments, Colorado Springs, CO, USA).

After the hemodynamic recording, the rat heart was removed to measure infarct size. A digital image of the left ventricle was captured by a digital camera (Canon, Japan). Infarct size was determined using a colorimetric technique coupled to a computerized planimetric analysis (Adobe Photoshop CS5 Extended). The percentage of infarct area to whole left ventricle was quantified using Adobe Photoshop CS5 Extended (Adobe Systems Incorporated, CA).

***Immunofluorescence staining***

The isolated SG was postfixed in 4% paraformaldehyde overnight and followed by soaking the heart in 30% sucrose for 12 hours at 4^o^C for cryostat protection. The SGs were cut into 10 μm-thick sections at -20^o^C and then mounted on pre-coated glass slides. Then the sections were incubated with 10% donkey serum for 1 hour followed by incubation with rabbit against TNFα antibody (ab9755, abcam, Cambridge, MA), rabbit against IL-1β antibody (ab9787, abcam, Cambridge, MA), goat anti-Iba1a antibody (a macrophage activation marker, ab178847, Abcam, Cambridge, MA), or mouse anti-tyrosine hydroxylase (TH) antibody (an adrenergic neuronal marker, T2928, Sigma-Aldrich, St. Louis, MO) overnight at 4°C. Then the sections were incubated with appropriate fluorescence-conjugated secondary antibodies (Invitrogen, Carlsbad, CA) for 60 min at room temperature. Slides were observed under a laser scanning confocal microscope (Zeiss LAM 710).

***Western blot analysis***

SGs were rapidly removed, immediately frozen in liquid nitrogen, and stored at –80°C until analyzed. Proteins in SG homogenates were extracted with a lysing buffer (10 mM Tris, 1 mM EDTA, 1% SDS; pH 7.4) plus protease inhibitor cocktail (100 µl ml-1, Sigma). After centrifugation at 12,000 g for 20 min at 4°C, the protein concentration in the supernatant was determined using a bicinchoninic acid protein assay kit (Pierce, Rockford, IL). Protein samples were mixed with the same volume of the loading buffer and heated for 5 min at 100°C. An equal amount of protein samples (40 μg/well) was loaded and then separated on a 9% sodium dodecyl sulfate (SDS)-polyacrylamide gel. Proteins of these samples were electrophoretically transferred at 200 mA for 3 hours onto PVDF membrane (EMD Millipore, Billerica, MA). The membrane was blocked with 5% non-fat milk in Tris-buffered saline-tween 20 (20 mM Tris, 150 mM NaCl, 0.05% (v/v) Tween-20) for 1 hour. Then the membrane was probed with rabbit primary antibody against TNFα (ab9755, abcam, Cambridge, MA), rabbit primary antibody against IL-1β (ab9787, abcam, Cambridge, MA), or goat anti-Iba1a antibody (a macrophage activation marker, ab178847, abcam, Cambridge, MA) overnight at 4⁰C. After washing, the membrane was incubated for 1 hour with peroxidase-conjugated appropriate secondary antibody (Pierce Chemical, Rockford, IL). The signal was detected using enhanced chemiluminescence substrate (Pierce Chemical, Rockford, IL). The blot was reprobed with mouse anti-β-actin or -GAPDH antibody. All bands were analyzed using UVP bioimaging system (UVP, Upland, CA). Target protein was normalized by a housekeeping protein.

***Cytokine array***

The levels of cytokines in SGs were detected by a Rat Cytokine Antibody Array C1 kit according to the User Manual (RayBiotech, Peachtree Corners, GA). SGs were rapidly removed, immediately frozen in liquid nitrogen, and stored at –80°C until analyzed. Proteins in SG homogenates were extracted with a lysing buffer (10 mM Tris, 1 mM EDTA, 1% SDS; pH 7.4) plus protease inhibitor cocktail (100 µl ml-1, Sigma). After centrifugation at 12,000 g for 20 min at 4°C, the protein concentration in the supernatant was determined using a bicinchoninic acid protein assay kit (Pierce, Rockford, IL). Protein samples were diluted (1× Blocking Buffer) to a final concentration of 100 µg/ml. After 2 ml of blocking buffer was added to each array membrane for 1-hour incubation at room temperature, 1 ml of diluted sample was added to the membrane and incubated overnight at 4 °C. After the membrane was washed with buffer I and II, 1 ml of diluted Biotin-conjugated antibody was added to incubate for 1 hour at room temperature. The membrane was washed again, then 2 ml of infrared fluorescent dye-conjugated streptavidin was added to incubate for 1 hour. The washed membrane was scanned on the LI-COR Odyssey infrared image system.

***Flow cytometry analyses for macrophage infiltration***

Flow cytometry is a widely used approach for measuring macrophage infiltration or expansion in many studies [12, 13, 21]. Here, SGs were enzymatically digested in 0.1% collagenase for 1 hour at 37°C. Single-cell suspensions of SGs were generated by a 35µm cell strainer. An aliquot of cell suspension was used for counting to derive the absolute number of cells in each sample. Dead cells were excluded using a Live/Dead Fixable Blue Dead Cell Stain Kit 30 min at 4°C. Cells were stained on ice for 20 min with Rat Fcblock^TM^, followed by incubation with a mix of fluorochrome-conjugated antibodies against CD45 and CD11b for 1 hour at 4°C. Cells were washed and resuspended in FACS buffer and run through a LSR‖ cell analyzer (BD Biosciences). Samples were analyzed with FlowJo software. Macrophages were identified as CD45^+^/CD11b^+^ cells [5, 8, 19].

***Echocardiography***

Echocardiography was performed to determine cardiac function [26]. Rats were imaged using the MX201 transducer (15 MHz) on the Vevo 3100 ultrasound machine (VisualSonics, Toronto, ON, Canada). Briefly, B-mode images were acquired in the parasternal long axis under light isoflurane anesthesia (1-1.5%). M-mode images were acquired at the level of the left ventricular papillary muscles. Left ventricular end-diastolic diameter (LVDd) and left ventricular end-systolic diameter (LVDs) were measured. Then, ejection fraction (EF), fractional shortening (FS), left ventricular end-diastolic volume (LVd Vol), and left ventricular end-systolic volume (LVs Vol) were calculated using standard formulas from the VisualSonics VevoLab software.

**Supplemental Figures and Figure legends**

**Supplemental Figure 1.**

**Supplemental Figure 1.** Study design, timeline, and interventions. At the beginning of these experiments, rats were randomly assigned to sham or CHF group. CHF rats underwent surgical LAD ligation to induce myocardial infarction (MI)-related CHF, and sham rats underwent the same surgery without LAD ligation. Implantation of radiotelemetry and labeling of cardiac sympathetic postganglionic (CSP) neurons were performed at 11 weeks post-MI. CHF rats were further assigned to three subgroups for different treatments, including CHF, CHF treated with PBS liposomes, and CHF treated with clodronate liposomes at 12 weeks post-MI. PBS liposomes or clodronate liposomes were microinjected into bilateral stellate ganglia (SGs). Heart rate variability (HRV), ventricular arrhythmogenesis-related ECG makers, and spontaneous ventricular arrhythmias from 24-hour radiotelemetry ECG recording in conscious rats were evaluated in all groups. Terminal experiments including measurement for inducibility of ventricular arrhythmia and cardiac sympathetic nerve activity (CSNA) recording in anesthetized rats, hemodynamic and morphological measurements, echocardiography, Western blot, immunofluorescence staining, cytokine array, flow cytometry, and whole-cell patch-clamp recording in CSP neurons were performed at 13-14 weeks post-MI. Survival surgeries for induction of CHF (or sham surgery) and drug treatment in SGs (clodronate liposomes, PBS liposomes, or vehicle) were performed in all rats. Survival surgeries for implantation of radiotelemetry and labeling of CSP neurons and non-survival surgeries for inducibility of ventricular arrhythmia and CSNA recording were separately performed in different rats.

**Supplemental Figure 2.**

**Supplemental Figure 2.** Cytokine levels in SGs from sham and CHF rats. A: Cytokine protein array map. B and C: Raw and quantitative data for cytokine protein array in SGs from sham and CHF rats. Levels of tumor necrosis factor alpha (TNFα) and interleukin-1 beta (IL-1β) in SGs were elevated in CHF rats, compared to sham rats. Data are means ± SEM; n=4 rats per group. Statistical significance was determined by a student’s unpaired t-test. *P<0.05 vs. sham. CINC: cytokine-induced neutrophil chemoattractants; GM-CSF: granulocyte-macrophage colony-stimulating factor; IL-1α: interleukin-1 alpha; IL-10: interleukin-10; LIX: lipopolysaccharide-induced CXC chemokine; VEGF: vascular endothelial growth factor.

**Supplemental Figure 3.**

**Supplemental Figure 3.** The effects of macrophage depletion in SG on total calcium currents elicited by 500-ms test pulse at 0 mV from holding potential of -80 mV (A), other types (including L-type, P/Q-type, and R-type) of Ca^2+^ currents elicited by 500-ms test pulse at 0 mV from holding potential of -80 mV (B), cell membrane capacitance (C), input resistance (D), and resting membrane potential (RMP, E) in cardiac sympathetic postganglionic (CSP) neurons from CHF rats. PBS liposomes or clodronate liposomes were *in-vivo* microinjected into the bilateral SGs under anesthesia. Data are means ± SEM; n=8 neurons from 6 rats per group. Statistical significance was determined by one-way ANOVA with post-hoc Bonferroni test. *P<0.05 vs. sham; ^†^p<0.05 vs. CHF.

**Supplemental Figure 4.**

**Supplemental Figure 4.** Macrophage depletion with clodronate liposomes in SGs attenuated CHF-elevated heterogeneity of ventricular electrical activities, which was calculated from 24-hour ECG recording in conscious rats. A: Representative tracings for QT and Tpe intervals in sham (upper) and CHF (lower) rats. B to F: Quantitative data for QT interval (B), QTc interval (C), QT dispersion (D), QTc dispersion (E), and Tpe (F) in all groups. Treatment with clodronate liposomes in SGs significantly inhibited CHF-induced prolongation of QT and QTc intervals, increases in QT and QTc dispersions, and elongation of Tpe interval. Data are means ± SEM; n=6 rats per group. Statistical significance was determined by one-way ANOVA with post-hoc Bonferroni test. *P<0.05 vs. sham; ^†^p<0.05 vs. CHF.

**Supplemental References**

1. Antzelevitch C (2007) Heterogeneity and cardiac arrhythmias: an overview. Heart Rhythm 4:964-972 doi:10.1016/j.hrthm.2007.03.036

2. Baltogiannis GG, Tsalikakis DG, Mitsi AC, Hatzistergos KE, Elaiopoulos D, Fotiadis DI, Kyriakides ZS, Kolettis TM (2005) Endothelin receptor--a blockade decreases ventricular arrhythmias after myocardial infarction in rats. Cardiovasc Res 67:647-654 doi:10.1016/j.cardiores.2005.04.020

3. Belichard P, Savard P, Cardinal R, Nadeau R, Gosselin H, Paradis P, Rouleau JL (1994) Markedly different effects on ventricular remodeling result in a decrease in inducibility of ventricular arrhythmias. J Am Coll Cardiol 23:505-513 doi:10.1016/0735-1097(94)90440-5

4. Carnevali L, Vacondio F, Rossi S, Macchi E, Spadoni G, Bedini A, Neumann ID, Rivara S, Mor M, Sgoifo A (2015) Cardioprotective effects of fatty acid amide hydrolase inhibitor URB694, in a rodent model of trait anxiety. Sci Rep 5:18218 doi:10.1038/srep18218

5. Chen Z, Feng X, Herting CJ, Garcia VA, Nie K, Pong WW, Rasmussen R, Dwivedi B, Seby S, Wolf SA, Gutmann DH, Hambardzumyan D (2017) Cellular and Molecular Identity of Tumor-Associated Macrophages in Glioblastoma. Cancer Res 77:2266-2278 doi:10.1158/0008-5472.CAN-16-2310

6. Costa EC, Goncalves AA, Areas MA, Morgabel RG (2008) Effects of metformin on QT and QTc interval dispersion of diabetic rats. Arq Bras Cardiol 90:232-238 doi:10.1590/s0066-782x2008000400004

7. Gui L, Bao Z, Jia Y, Qin X, Cheng ZJ, Zhu J, Chen QH (2013) Ventricular tachyarrhythmias in rats with acute myocardial infarction involves activation of small-conductance Ca2+-activated K+ channels. Am J Physiol Heart Circ Physiol 304:H118-130 doi:10.1152/ajpheart.00820.2011

8. Hawkins KE, DeMars KM, Alexander JC, de Leon LG, Pacheco SC, Graves C, Yang C, McCrea AO, Frankowski JC, Garrett TJ, Febo M, Candelario-Jalil E (2017) Targeting resolution of neuroinflammation after ischemic stroke with a lipoxin A4 analog: Protective mechanisms and long-term effects on neurological recovery. Brain Behav 7:e00688 doi:10.1002/brb3.688

9. Hong T, Yang H, Zhang SS, Cho HC, Kalashnikova M, Sun B, Zhang H, Bhargava A, Grabe M, Olgin J, Gorelik J, Marban E, Jan LY, Shaw RM (2014) Cardiac BIN1 folds T-tubule membrane, controlling ion flux and limiting arrhythmia. Nat Med 20:624-632 doi:10.1038/nm.3543

10. Jeong SW, Wurster RD (1997) Calcium channel currents in acutely dissociated intracardiac neurons from adult rats. J Neurophysiol 77:1769-1778 doi:10.1152/jn.1997.77.4.1769

11. Kang CS, Chen CC, Lin CC, Chang NC, Lee TM (2009) Effect of ATP-sensitive potassium channel agonists on sympathetic hyperinnervation in postinfarcted rat hearts. Am J Physiol Heart Circ Physiol 296:H1949-1959 doi:10.1152/ajpheart.00903.2008

12. Lindborg JA, Niemi JP, Howarth MA, Liu KW, Moore CZ, Mahajan D, Zigmond RE (2018) Molecular and cellular identification of the immune response in peripheral ganglia following nerve injury. J Neuroinflammation 15:192 doi:10.1186/s12974-018-1222-5

13. Liu L, Yin Y, Li F, Malhotra C, Cheng J (2017) Flow cytometry analysis of inflammatory cells isolated from the sciatic nerve and DRG after chronic constriction injury in mice. J Neurosci Methods 284:47-56 doi:10.1016/j.jneumeth.2017.04.012

14. Murakami M, Niwa H, Kushikata T, Watanabe H, Hirota K, Ono K, Ohba T (2014) Inhalation anesthesia is preferable for recording rat cardiac function using an electrocardiogram. Biol Pharm Bull 37:834-839 doi:10.1248/bpb.b14-00012

15. Nguyen T, El Salibi E, Rouleau JL (1998) Postinfarction survival and inducibility of ventricular arrhythmias in the spontaneously hypertensive rat : effects of ramipril and hydralazine. Circulation 98:2074-2080 doi:10.1161/01.cir.98.19.2074

16. Opitz CF, Mitchell GF, Pfeffer MA, Pfeffer JM (1995) Arrhythmias and death after coronary artery occlusion in the rat. Continuous telemetric ECG monitoring in conscious, untethered rats. Circulation 92:253-261 doi:10.1161/01.cir.92.2.253

17. Pardini BJ, Patel KP, Schmid PG, Lund DD (1987) Location, distribution and projections of intracardiac ganglion cells in the rat. J Auton Nerv Syst 20:91-101 doi:10.1016/0165-1838(87)90106-8

18. Rossi S, Fortunati I, Carnevali L, Baruffi S, Mastorci F, Trombini M, Sgoifo A, Corradi D, Callegari S, Miragoli M, Macchi E (2014) The effect of aging on the specialized conducting system: a telemetry ECG study in rats over a 6 month period. PLoS One 9:e112697 doi:10.1371/journal.pone.0112697

19. Savarin C, Dutta R, Bergmann CC (2018) Distinct Gene Profiles of Bone Marrow-Derived Macrophages and Microglia During Neurotropic Coronavirus-Induced Demyelination. Front Immunol 9:1325 doi:10.3389/fimmu.2018.01325

20. Shiba Y, Fernandes S, Zhu WZ, Filice D, Muskheli V, Kim J, Palpant NJ, Gantz J, Moyes KW, Reinecke H, Van Biber B, Dardas T, Mignone JL, Izawa A, Hanna R, Viswanathan M, Gold JD, Kotlikoff MI, Sarvazyan N, Kay MW, Murry CE, Laflamme MA (2012) Human ES-cell-derived cardiomyocytes electrically couple and suppress arrhythmias in injured hearts. Nature 489:322-325 doi:10.1038/nature11317

21. Simeoli R, Montague K, Jones HR, Castaldi L, Chambers D, Kelleher JH, Vacca V, Pitcher T, Grist J, Al-Ahdal H, Wong LF, Perretti M, Lai J, Mouritzen P, Heppenstall P, Malcangio M (2017) Exosomal cargo including microRNA regulates sensory neuron to macrophage communication after nerve trauma. Nat Commun 8:1778 doi:10.1038/s41467-017-01841-5

22. Tu H, Liu J, Zhang D, Zheng H, Patel KP, Cornish KG, Wang WZ, Muelleman RL, Li YL (2014) Heart failure-induced changes of voltage-gated Ca2+ channels and cell excitability in rat cardiac postganglionic neurons. Am J Physiol Cell Physiol 306:C132-142 doi:10.1152/ajpcell.00223.2013

23. Yagishita D, Chui RW, Yamakawa K, Rajendran PS, Ajijola OA, Nakamura K, So EL, Mahajan A, Shivkumar K, Vaseghi M (2015) Sympathetic nerve stimulation, not circulating norepinephrine, modulates T-peak to T-end interval by increasing global dispersion of repolarization. Circ Arrhythm Electrophysiol 8:174-185 doi:10.1161/CIRCEP.114.002195

24. Yan GX, Lankipalli RS, Burke JF, Musco S, Kowey PR (2003) Ventricular repolarization components on the electrocardiogram: cellular basis and clinical significance. J Am Coll Cardiol 42:401-409 doi:10.1016/s0735-1097(03)00713-7

25. Zhang D, Liu J, Tu H, Muelleman RL, Cornish KG, Li YL (2014) In vivo transfection of manganese superoxide dismutase gene or nuclear factor kappaB shRNA in nodose ganglia improves aortic baroreceptor function in heart failure rats. Hypertension 63:88-95 doi:10.1161/HYPERTENSIONAHA.113.02057

26. Zhang D, Tu H, Wang C, Cao L, Hu W, Hackfort BT, Muelleman RL, Wadman MC, Li YL (2021) Inhibition of N-type calcium channels in cardiac sympathetic neurons attenuates ventricular arrhythmogenesis in heart failure. Cardiovasc Res 117:137-148 doi:10.1093/cvr/cvaa018

27. Zhang D, Tu H, Wang C, Cao L, Muelleman RL, Wadman MC, Li YL (2017) Correlation of Ventricular Arrhythmogenesis with Neuronal Remodeling of Cardiac Postganglionic Parasympathetic Neurons in the Late Stage of Heart Failure after Myocardial Infarction. Front Neurosci 11:252 doi:10.3389/fnins.2017.00252
